# Testing Effects of Foreign Language Listening Anxiety on Chinese University Students' English Listening Test Performance

**DOI:** 10.3389/fpsyg.2021.701926

**Published:** 2021-07-12

**Authors:** Meihua Liu, Hongliang Xu

**Affiliations:** ^1^Department of Foreign Languages and Literatures, Tsinghua University, Beijing, China; ^2^School of Foreign Studies, Fuyang Normal University, Fuyang, China

**Keywords:** foreign language listening anxiety, test performance, English proficiency, gender, effect

## Abstract

The present research explored how foreign language listening anxiety (FLLA) affected Chinese university students' English listening test performance and how proficiency and gender mediated the effects of FLLA on the latter. Two different populations from two universities in China answered the 20-item Foreign Language Listening Anxiety Scale (FLLAS) as well as a demographic questionnaire and took an English listening test. Analyses of the collected data revealed the following major findings: (a) Five latent factors underlay the FLLAS, (b) when working alone, FLLA significantly negatively predicted students' English listening test performance, and (c) when working with proficiency and gender, English proficiency level, gender and FLLAS2 (proficiency in English listening) significantly predicted students' English listening test performance. These findings confirm the negative effects of FLLA on students' English listening test performance. They also indicate that English proficiency and gender mediate FLLA's effects on the latter, with English proficiency not only directly but also indirectly affecting the latter.

## Introduction

Foreign language anxiety (FLA), an important psychological and affective variable, has been widely researched in second language acquisition and proven to be an influencing factor for second/foreign language learning outcomes (e.g., Horwitz et al., 1986; MacIntyre and Gardner, 1994). Even so, the current literature shows that most FLA research focuses on speaking which is generally acknowledged to be the most anxiety-provoking activity in second/foreign language learning (Horwitz et al., 1986; MacIntyre and Gardner, 1994; Liu, 2016). Other types of FLA such as listening anxiety, reading anxiety and speaking anxiety, though also existent in second/foreign language learning, has generally been under-researched (e.g., Elkhafaifi, 2005; Liu and Thondhlana, 2015). In particular, often intertwined with speaking, listening is also a very challenging and stressful task for second/foreign language learners (Arnold, 2000). Although several questionnaires have been developed to measure foreign language listening anxiety (Elkhafaifi, 2005; Yamauchi, 2014), they need to be validated or analyzed to identify their underlying components to better understand their effects on learning listening to a second/foreign language. Hence, the present research aims to explore the latent factors underlying the Foreign Language Listening Anxiety Scale used in Elkhafaifi (2005). It also seeks to test how foreign language listening anxiety (FLLA) affects Chinese university students' English listening test performance and how English proficiency and gender mediate the effects of FLLA on the latter.

## Literature Review

As defined by MacIntyre and Gardner (1994, p.5), foreign language anxiety is the “apprehension experienced when a situation requires the use of a second language with which the individual is not fully proficient”. It refers to the nervous reactions when speaking, listening to, reading, or writing in the second/foreign language (Horwitz et al., 1986; MacIntyre and Gardner, 1994). Since the 1970s, a multitude of research has been done on FLA in various contexts of both traditional classroom and online learning situations, which generally reveals that FLA is experienced by many learners and predominantly debilitates their learning of a second/foreign language (Horwitz et al., 1986; Xiangming et al., 2020; Liu, 2021).

The current literature also shows that FLA exists in all aspects of second/foreign language learning such as listening, speaking, reading and writing, although speaking is generally endorsed to be the most anxiety-provoking activity in second/foreign language learning and hence has been most researched (Horwitz et al., 1986; Gregersen, 2020). Nevertheless, research on these types of FLA is rather inadequate (Elkhafaifi, 2005; Liu, 2016), which shows that they are distinct and independent constructs. Due to such factors as task unfamiliarity, incomprehensibility, and fear of embarrassing outcomes (Elkhafaifi, 2005), many learners tend to become anxious when listening to a second/foreign language (Arnold, 2000; Zhang, 2013; Liu, 2016). To measure this anxiety, Elkhafaifi (2005) developed the 20-item Foreign Language Listening Anxiety Scale (FLLAS) and administered it and a foreign language learning anxiety scale to 233 post-secondary students of Arabic as a foreign language. He found that both general foreign language learning anxiety and listening anxiety negatively affected students' listening comprehension and their final grades. This finding is generally supported by research adopting the FLLAS or other methods like interviews (Chang, 2008; Bekleyen, 2009; Golchi, 2012; Serraj and Noordin, 2013; Zhang, 2013; Wang and Cha, 2019).

To analyze the FLLAS's latent components, Zhang (2013) ran structural equation modeling on the FLLAS and revealed three dimensions: five-item general anxiety about English listening indicating nervousness, upset/distress, or feeling intimidated when facing listening activities, three-item self-belief involving confidence and satisfaction with one's foreign language listening proficiency, and three-item listening decoding skills concerned with learners' cognitive ability related to memory, attention and understanding. These three components were then used in Liu (2016; Liu and Thondhlana, 2015) together with foreign language listening strategy use, which found that FLLA significantly negatively predicted students' English listening comprehension. The study also revealed that foreign language listening anxiety and strategy use were significantly correlated with each other and that proficiency greatly mediated students' FLLA levels and use of foreign language listening strategies. Similarly, Wang and Cha's (2019) study of 78 Chinese university students showed that English proficiency mediated the effect of FLLA on students' listening performance. In addition, Liu and Thondhlana (2015) found that male students were significantly more anxious about English listening and less satisfied with their English listening proficiency than their female peers. Meanwhile, Dewaele's (2017) study of 1287 female and 449 male students revealed that females had more fun and anxiety in the foreign language class. MacIntyre et al. (2002) and Hasan and Fatimah (2014) found that male students experienced greater anxiety than their female counterparts when speaking English. By contrast, Hwa's (2014) exploration of 237 Malaysian undergraduate students showed that female students were significantly more anxious than their male peers when speaking English. Evidently, gender mediates foreign language anxiety in second/foreign language learning. It is likely to mediate FLLA as well, which needs to be researched.

## Research Questions

As reviewed above, foreign language listening anxiety is much under-researched though it exists in second/foreign learning. Meanwhile, though the Foreign Language Listening Anxiety Scale (FLLAS) has been analyzed (Zhang, 2013) and used in empirical studies (Liu and Thondhlana, 2015; Liu, 2016), the revealed latent components fail to cover all the items. Moreover, as shown in Table 1, the FLLAS items generally reflect different aspects of anxiety related to listening to English. For example, item 1 “I get upset when I'm not sure whether I understand what I'm hearing in English” concerns anxiety about listening to English; item 2 “When I listen to English, I often understand the words but still can't quite understand what the speaker is saying” involves proficiency in English listening. It is thus necessary to explore again the factors underlying the FLLAS and investigate their effects on second/foreign language learner's listening performance. In addition, several studies reveal that proficiency mediates students' FLLA levels or the effect of FLLA on their listening performance (Liu, 2016; Wang and Cha, 2019). Gender is also found to affect the levels of foreign language anxiety and its effect on second/foreign language learning outcomes (Liu and Thondhlana, 2015; Liu, 2021). Hence, it is also worthwhile to investigate how proficiency and gender mediate the effects of FLLA on students' listening performance in a second/foreign language. Targeting Chinese undergraduate students, the present study formulated the following research questions:

1. What are the factors underlying the Foreign Language Listening Anxiety Scale?
2. How does FLLA affect Chinese university students' English listening test performance?
3. How do students' English proficiency and gender mediate FLLA's effects on their English listening test performance?

**Table 1:** Loadings of Principal Components of FLLAS in Study 1 and Study 2.

	**FLLAS1**	**FLLAS2**	**FLLAS3**	**FLLAS4**	**FLLAS5**
1. I get upset when I'm not sure whether I understand what I'm hearing in English.	**0.465/0.678**	0.466/0.065	0.027/0.183	0.092/0.016	−0.054/0.092
2. When I listen to English, I often understand the words but still can't quite understand what the speaker is saying.	0.138/0.079	**0.675/0.068**	0.082/0.854	−0.043/0.050	0.076/0.009
3. When I'm listening to English, I get so confused I can't remember what I've heard.	0.286/0.382	0.669/0.144	**0.076/0.648**	0.164/0.137	−0.053/0.116
4. I feel intimidated whenever I have a listening passage in English to listen to.	**0.607/0.603**	0.314/0.154	0.174/0.238	0.177/0.301	0.058/-0.031
5. I am nervous when I am listening to a passage in English when I'm not familiar with the topic.	**0.647/0.754**	0.365/0.078	0.047/0.229	0.092/0.072	−0.079/0.046
6. I get upset whenever I hear unknown grammar while listening to English.	**0.789/0.785**	0.035/0.044	0.067/0.135	0.058/0.087	0.124/−0.005
7. When listening to English I get nervous and confused when I don't understand every word.	**0.784/0.725**	0.107/0.227	0.096/0.046	0.112/0.102	−0.095/−0.116
8. It bothers me to encounter words I can't pronounce while listening to English.	**0.674/0.684**	0.167/0.117	0.120/0.069	0.134/0.166	0.032/−0.155
9. I usually end up translating word by word when I'm listening to English.	0.459/0.288	0.225/0.065	**0.291/0.417**	0.155/0.146	0.230/−0.170
10. By the time I get past the strange sounds in English, it's hard to remember what I'm listening to.	0.318/0.385	0.517/0.270	**0.141/0.432**	0.045/−0.082	−0.008/0.058
11. I am worried about all the new sounds I have to learn to understand spoken English.	**0.196/0.298**	0.310/0.164	0.032/0.188	0.544/0.478	0.213/0.178
12. I enjoy listening to English.	0.142/0.142	0.195/0.754	0.751/0.064	**0.066/0.249**	0.016/−0.204
13. I feel confident when I am listening to English.	**0.141/0.214**	0.254/0.794	0.706/0.219	−0.037/0.033	−0.240/−0.103
14. Once I get used to it, listening to English is not so difficult.	0.106/0.162	**0.075/0.483**	0.611/0.013	0.078/0.318	0.405/−0.444
15. The hardest part of learning English is learning to understand spoken English.	0.000/-0.080	**0.117/0.057**	−0.075/0.117	0.724/0.298	−0.318/0.650
16. I would be happy just to learn to read English rather than having to learn to understand spoken English.	0.194/-0.007	−0.153/−0.042	0.064/−0.097	**0.667/0.773**	0.184/0.130
17. I don't mind listening to English by myself but I feel very uncomfortable when I have to listen to English in a group.	**0.371/0.252**	0.055/0.136	0.125/0.149	0.419/0.462	0.087/0.003
18. I am satisfied with the level of listening comprehension in English that I have achieved so far.	0.079/0.122	**0.364/0.623**	0.212/0.076	0.066/−0.311	−0.665/0.418
19. English culture and ideas seem very foreign to me.	0.102/0.167	0.316/−0.010	0.096/0.299	0.157/0.431	**0.645/**–**0.290**
20. I have to know so much about English history and culture in order to understand spoken Arabic.	−0.085/0.047	0.351/−0.170	−0.508/−0.079	0.005/0.009	**0.060/0.645**

*The loadings of items and their corresponding factors are highlighted in bold*.

## Research Design

This paper reports on the results of an on-going project on affective and psychological variables and learning of English as a foreign language. In the present research, two studies were conducted on two student populations at different English proficiency levels with appropriate gender ratios from various disciplines of two universities in different parts of China. In both universities (A and B), undergraduate non-English majors took a standardized English placement test upon entering the university prior to the start of formal classroom teaching, the results of which placed them at different proficiency levels. University A put students into three proficiency levels (1–3) while university B divided students to only two levels (1 and 3). In both cases, a higher level meant higher proficiency in English and more students went to the level 1 group. Moreover, in both universities, undergraduate non-English majors had to take the College English Test band 4 (CET-4) to graduate on time, which is an exit and proficiency test administered two times a year to all undergraduate non-English majors nationwide. The written test consists of four parts: Writing (15%), listening (35%), reading (35%) and translation (15%). The listening part consists of three parts: Three short monologs (7%), two long dialogues (8%) and three long texts (20%), each followed by multiple-choice questions. Students can take the test any time in university and most tend to take it in their third year of study. A testee who gets 85% or higher in the written test can take a speaking test which is administered separately and later. Thus, an English Listening and Speaking course is generally required for first- and second-year non-English majors but optional for those in other years of study in most universities.

Both universities had two major terms of an academic year, yet university A had 16 weeks while university B had 18 weeks for each term. Both were state-owned and comprehensive universities. Being more prestigious, university A was renowned for science and technology and was predominantly male. It was more challenging to be admitted into university A as well, which often required its students to study hard and be good in study. University B was more balanced in the development of different disciplines and happened to have more female students. The participants of both studies for the present research were sampled from those registered in an English Listening and Speaking course in both universities. Detailed information about the participants in each study was presented in Table 2, which shows that study 1 and study 2 had contrasting gender ratios and that the respondents were at three different proficiency levels. Of 536 (371 male and 165 female) participants in study 1,409 (76.3%) were first-year students, 82 (15.3%) second-year and 44 (8.2%) third-year students, 198 (36.8%) were level 1 students, 201 (37.5%) level 2 and 138 (25.7%) level 3 students. Of 399 (82 male and 317 female) participants in study 2,200 (50.1%) were first-year and 199 (49.9%) second-year students, 223 (55.9%) were level 1 and 176 (44.1%) level 2 students. Table 2 also shows that the participants in both studies fell into a similar age range of 17–24, with those in study 1 having a larger age range. This was because the participants in study 1 were from the first-, second- and third-year of study respectively, while there were no third-year respondents in study 2, although more than half participants in both studies were year-1 students.

**Table 2:** Characteristics of the participants and the FLLAS.

	**Participants**										**The FLLAS (No**. **=** **20)**	
	**Sample size**		**Age**		**Year of study**			**English proficiency level**			**Reliability**	**MIT correlation**
	**M**	**F**	**Mean**	**Range**	**1**	**2**	**3**	**1**	**2**	**3**		
Study1	371	165	18.75	17–24	409/ 76.3%	82/15.3%	44/ 8.2%	197/36.8%	201/ 37.5%	138/25.7%	0.832	0.407
Study2	82	317	19.65	17–23	200/ 50.1%	199/49.9%		223/55.9%		176/44.1%	0.828	0.403

*M, male (1); F, female (2); MIT correlation, mean item-total correlation (p = 0.01)*.

The participants in both studies answered the 20-item Foreign Language Listening Anxiety Scale, the background questionnaire and took an English listening test.

### Foreign Language Listening Anxiety Scale

The 20-item Foreign Language Listening Anxiety Scale (FLLAS) used in Elkhafaifi (2005) was employed in the present research to test its effect on students' English listening test performance. To better suit the present study, the expression “Arabic” in the original FLLAS items was changed to be “English”. Placed on a 5-point Likert scale, each FLLAS item had five choices, ranging from “*strongly disagree*” to “*strongly agree*”, with values of 1–5 assigned to the choices respectively. The higher the score, the more anxious a respondent was. As presented in Table 2, the FLLAS had a reliability score range of 0.828 to 0.832 and mean item-total correlations ranging from 0.403 to 0.407 (*p* = 0.01) in the two studies, similar to the scores in Liu (2016).

### Background Information Questionnaire

The background information questionnaire aimed to collect such information about the participants as gender, age, discipline, year of study, and English proficiency levels.

### English Listening Test Performance

The participants' English listening test performance was measured by English listening tests they took in their respective courses. Serving as mid-term exams in university A and final-term exams in university B, the listening tests used in the two studies were designed by instructors of the same course to suit their students respectively and thus might be at varying difficulty levels. Yet they all simulated that in CET-4, had 100 points in total and consisted of three parts: Multiple-choice questions for 15 short dialogues (15%) and three longer conversations (40%), and dictation and multiple-choice questions for two essays of around 500 words (45%).

Study 1 was conducted in the middle of university A's 16-week term and study 2 in the last week of university B's 18-week term in the first term of the same academic year. In both studies, the students took the listening test first and then answered the questionnaire together with a consent form. The collected test scores and survey data were analyzed via SPSS 20. Principal components (varimax) factor analyses were run on the FLLAS to identify its underlying factors. Then, multiple regression analyses (stepwise) were conducted to examine the predicting effects of FLLAS and the mediating effects of English proficiency and gender on students' English listening test performance.

## Findings

### Factors Underlying the FLLAS

Prior to regression analyses, the FLLAS was subjected to rotated (varimax) principal components analysis in study 1 (Kaiser-Meyer-Olkin measure of sampling adequacy/KMO = 0.871, χ^2^ = 265.2, *N* = 537, *p* = 0.001) and study 2 (*KMO* = 0.864, χ^2^ = 215.8, *N* = 399, *p* = 0.001), both of which yielded five latent factors. The eigenvalues and loadings are displayed in Tables 3 and 1 respectively. With reference to Zhang (2013), the five factors were: nine-item FLLAS1 reflective of anxiety about listening to English, four-item FLLAS2 suggestive of proficiency in English listening, three-item FLLAS3 concerned with English listening decoding skills, two-item FLLAS4 indicative of liking for listening to English, and two-item FLLAS5 related to English culture in learning English listening.

**Table 3:** Eigenvalues and explained variances of FLLAS factors.

	**Initial Eigenvalues**		**Rotation Sum of Squared Loadings**	
**Factor**	**Total**	**% of Variance**	**Total**	**% of Variance**
FLLAS1	5.356/5.472	26.779/27.361	3.396/3.681	16.982/18.406
FLLAS2	1.562/1.639	7.809/8.196	2.313/2.104	11.563/10.519
FLLAS3	1.499/1.464	7.493/7.318	1.954/1.912	9.769/9.558
FLLAS4	1.155/1.312	5.777/6.558	1.619/1.765	8.093/8.824
FLLAS5	1.081/1.065	5.407/5.324	1.371/1.490	6.857/7.449

*FLLAS1, Anxiety about listening to English; FLLAS2, Proficiency in English listening; FLLAS3, English listening decoding skills; FLLAS4, Liking for listening to English; FLLA5, Culture in learning listening to English*.

*In each line, the first number is for study 1 and the second for study 2*.

### Predicting Effects of FLLA

To examine the predicting effects of FLLA on students' listening performance, listening test scores were used as the dependent variable and the FLLAS scales were used as independent variables when running multiple stepwise regression analyses in the two studies (see Table 4). The analyses yielded one model with the change in R^2^ being 0.013: model 1 (FLLAS3, β = −0.115, *t* = −2.684, *p* = 0.007, *f*^2^ = 0.013) for study 1, and one model with the change in R^2^ being 0.016: model 1 (FLLAS2, β = −0.127, *t* = −2.553, *p* = 0.011, *f*^2^ = 0.016) for study 2. Namely, FLLAS3 (English listening decoding skills) was a powerful negative predictor for students' English listening test performance in study 1 and FLLAS2 (proficiency in English listening) a powerful negative predictor for the latter in study 2.

**Table 4:** Regression coefficients and significance of predictors for english listening test performance (FLLAS scales as independent variables).

	**English listening test performance**				
Study 1	β	*t*	*p*	VIF	Cohen's f^*2*^
FLLAS3	−0.115	−2.684^**^	0.007	1.000	0.013
	**English listening test performance**				
Study 2	β	*t*	*p*	VIF	Cohen's f^*2*^
FLLAS2	−0.127	−2.553^*^	0.011	1.000	0.016

** *, p ≤ 0.01;*

* *, p ≤ 0.05; effect size of Cohen's f^2^: small, f^2^ ≤ 0.02; medium, f^2^ = 0.15; large, f^2^ ≥ 0.35 (Cohen, 1988)*.

To examine the mediating effects of English proficiency and gender on FLLA, listening test scores were used as the dependent variable, and the FLLAS scales, gender and English proficiency level were used as independent variables when running multiple stepwise regression analyses in studies 1 and 2. As reported in Table 5, the analyses yielded one model with the change in R^2^ being 0.071: model 1 (English proficiency level, β = 0.266, *t* = 6.288, *p* = 0.000, *f*^2^ = 0.071) for study 1. This meant that English proficiency level significantly positively predicted students' English listening test performance in study 1. The analyses revealed three models for study 2, with the change in R^2^ being 0.104 for model 1 (English proficiency level), 0.052 for model 2 (English proficiency level, gender), and 0.008 for model 3 (English proficiency level, gender, and FLLAS2). Model 3 proved to be the fittest: English proficiency level (β =0.326, *t* = 7.038, *p* = 0.000, *f*^2^ = 0.104), gender (β = 224, *t* = 4.854, *p* = 0.000, *f*^2^ = 0.078), and FLLAS2 (β = −0.092, *t* = −1.994, *p* = 0.047, *f*^2^ = 0.055) were powerful predictors for students' English listening test performance. Alternatively, generally with a medium effect size, English proficiency level and gender significantly positively while FLLAS2 (proficiency in English listening) significantly negatively predicted students' English listening test performance to.

**Table 5:** Regression coefficients and significance of predictors for english listening test performance (FLLAS, English proficiency and gender as independent variables).

	**English listening test performance to**				
Study 1	β	*t*	*p*	VIF	Cohen's f^*2*^
English proficiency level	0.266	6.288^**^	0.000	1.000	0.071
	**English listening test performance**				
Study 2	β	*t*	*p*	VIF	Cohen's f^*2*^
English proficiency level	0.326	7.038^**^	0.000	1.009	
gender	0.224	4.854^**^	0.000	1.005	
FLLAS2	−0.092	−1.994^*^	0.047	1.009	0.055

** *, p ≤ 0.01;*

* *, p ≤ 0.05; effect size of Cohen's f^2^: small, f^2^ ≤ 0.02; medium, f^2^ = 0.15; large, f^2^ ≥ 0.35 (Cohen, 1988)*.

## Discussion and Conclusion

The present study revealed five latent factors underlying the FLLAS: Anxiety about listening to English (FLLAS1), proficiency in English listening (FLLAS2), English listening decoding skills (FLLAS3), liking for listening to English (FLLAS4) and English culture (FLLAS5). These five factors reflected learners' worry about the activity of listening to English and their own proficiency in English listening, skills used to decode what was heard, attitudes toward listening to English and English culture involved in comprehending what was heard in English. All these were consistent with the definition that FLLA refers to the feeling of worry and apprehension resultant from listening-related activities (Zhang, 2013; Liu, 2016). Nevertheless, this finding was different from Zhang's (2013), mainly due to the fact that Zhang's three factors did not cover all the 20 items. Since, FLLAS items reflective of liking for listening to English and English culture needed to learn English listening well are distinct in meaning, they should be clustered on their own to indicate respective dimensions of the overall FLLAS. The identification of these factors helps us better understand which dimension makes listeners anxious about listening to the target language and affects their performance in listening to the language. All these yet need to be further supported with data collected from varying learner populations.

When working alone, FLLAS3 (English listening decoding skills) proved to be a powerful negative predictor for students' English listening test performance in study 1 and FLLAS2 (proficiency in English listening) a powerful negative predictor for the latter in study 2, as found in Liu (2016). Alternatively, in study 1, failure to process or decode the listening input (e.g., failing to remember what was heard due to confusion or strange sounds, translating word by word) powerfully predicted students' poor English listening test performance. In study 2, low proficiency in English listening (e.g., inability to understand the speaker, difficulty of English listening) powerfully negatively predicted students' English listening test scores. This difference might be due to the characteristics of the participants in the two studies. As reported in Table 2, though the respondents in study 1 were largely evenly distributed among the three proficiency levels, the difference between different levels might not be big since the respondents were primarily first-year students. Coupled with the fact that their university was more prestigious and more challenging, they were probably more proficient in English and more confident about their English proficiency. Consequently, decoding skills instead of proficiency significantly predicted their' listening test performance. Contrarily, the participants of study 2 were nearly evenly divided between first- and second-year students and between level 1 and level 3 groups, which made proficiency a marked predictor for students' listening test performance.

When working with English proficiency level and gender, no FLLAS factor was found to significantly predict students' English listening test performance, while English proficiency level proved to significantly positively predict the latter in study 1. In study 2, English proficiency level and gender significantly while FLLAS2 (proficiency in English listening) significantly negatively predicted the latter. The different effects demonstrated by gender might be due to the contrasting yet different male to female rations in study 1 (371/165 = 2.25/1) and study 2 (82/317 = 1/3.87). In general, these findings confirmed the finding that foreign language listening anxiety inversely affects students' English listening test performance (Elkhafaifi, 2005; Zhang, 2013; Liu and Thondhlana, 2015; Liu, 2016, 2021; Wang and Cha, 2019). Meanwhile, they indicated that English proficiency level and gender mediated FLLA's effects on students' English listening test performance, as found in Liu (2016, 2021) and Wang and Cha (2019). Coupled with the fact that a higher FFLAS2 score meant lower proficiency in English, these findings also implied that English proficiency could not only directly affect learners' English listening test performance but mediate FLLA to affect it indirectly.

The present research also revealed that FLLAS4 (liking for listening to English) and FLLAS5 (English culture) did not predict the participants' English listening test performance in either case. This might be because liking was not equal to real efforts invested to learn English listening or the ability to well understand what was heard in English. Meanwhile, since the course was primarily for first- and second-year students, the listening materials might not involve much unfamiliar English culture which would affect students' comprehension. However, for similar reasons, FLLAS4 and FLLAS5 might exert effects on students' English listening (test) performance in other contexts, especially when the listening materials are loaded with culture of the target language. This further justifies more and continuous research on foreign language listening anxiety.

The present research did not reveal that anxiety about the activity of listening to English (FLLAS1) was a predictor of students' English listening test performance. Nevertheless, as foreign language listening anxiety might result from unfamiliarity with words, topics or tasks, incomprehensibility, and worry about outcomes (Elkhafaifi, 2005), the findings of this research clearly showed that anxiety did negatively affect students' English listening test performance to, similar to the finding in related studies of the current literature (Elkhafaifi, 2005; Yamauchi, 2014; Liu and Thondhlana, 2015; Liu, 2016; Wang and Cha, 2019). Even so, as discussed in Liu (2016), students had better pay special attention to the strategies of processing and decoding the input while listening to English. It may be more helpful to use such strategies as guessing and predicting instead of translating word by word (Roussel, 2011). This can be done in explicit or implicit classroom teaching and learning activities (Maleki, 2007). For example, Chang (2008) found that students who previewed questions and listened to the same material repeatedly became significantly less anxious and performed better when listening to English. Yamauchi (2014) found that FLLA might be from lack of knowledge, difficulty of the material, inappropriate processing of the spoken input, and listeners' metacognitive activities. Thus, students are advised to maximize their exposure to and practice of listening to English spoken with varying accents at differing speeds. By getting used to various accents and speech rates, students may find it easier to process, decode and comprehend the spoken input while listening to English. Likewise, it is important for students to improve their proficiency in English, which is the primary source of confidence and anxiety in learning and using the target language in any form (Horwitz et al., 1986; Liu, 2016; Wang and Cha, 2019).

Despite the revealing findings, some limitations existed in the research. As shown in Table 2, the present research gathered data from first-, second- and third-year students in study 1 but happened to collect no data from third-year students in study 2. This might be because few third-year students took the English Listening and Speaking course in university B when the research was conducted. Yet, although third-year students only accounted for a small percent (8.2%) of the total sample of study 1 and did not make significant difference to the general findings, an inclusion of some third-year students in study 2 as well would have better reflected the diversity of the populations studied in the research. This, together with the absence of level 2 in study 2, should be carefully treated in future research. Future studies should also be cautious about the effects of other factors such as age and year of study, which might mediate FLLA to varying degrees if researched.

## Data Availability Statement

The raw data supporting the conclusions of this article will be made available by the authors, without undue reservation.

## Ethics Statement

The studies involving human participants were reviewed and approved by Research ethics committee, Department of Foreign Languages and Literatures, Tsinghua University. The patients/participants provided their written informed consent to participate in this study.

## Author Contributions

ML designed the research, analyzed the data, wrote the Results and Discussion part, and proofread the manuscript. HX collected the data, wrote the other parts and proofread the manuscript. All authors contributed to the article and approved the submitted version.

## Conflict of Interest

The authors declare that the research was conducted in the absence of any commercial or financial relationships that could be construed as a potential conflict of interest.
